# Illustrations of Certain Points in the History of Gout

**Published:** 1855-04

**Authors:** 


*Medical Society of London, Feb.* 7, 1855.

*Illustrations of Certain Points in the History of Gout.*—Dr. Garrod, in his paper, proceeded to consider the chronic states of the articular and non-articular forms of gout. He said, that difficult as it was to separate some forms of acute gout from acute rheumatism, yet this was more difficult still in their chronic state. As to the symptoms which might be brought to bear upon this diagnosis, he believed that it would be allowed by all that the chalk-like deposits, or chalk-stones, are never found except in the subjects of true gout. In composition these deposits consist essentially of urate of soda, the difference met with in the analyses being dependent on the tissues in which they occur; they are alkaline in reaction, and, at first, fluid, going through different degrees of consistence till they become solid. The situations in which they are found, vary exceedingly. They are found within and around the joints, around the ligaments and sheaths of tendons, on the surface of the cartilage of the joints, and under the cuticle, etc. When occurring within the joints, they produce a weightiness on the cartilage, and greatly impede motion. They may remain in the body during the lifetime of the patient, which, probably, is generally the case; or they may be dischargod by a kind of desquamation, when, more deeply seated, by causing inflammation or ulceration. When these deposits occur where they may be readily seen,—as, for instance, around the joints, the diagnosis of the case is easily made; but they are not unfrequently confined to a single part of the body, and then are likely to be overlooked by the practitioner. The ear, and sometimes the integuments of the face, are the parts most commonly selected externally, where they may exist singly, or in numbers. They exist more frequently in the ears than has been generally supposed; and it was ascertained, from a collection of cases made, that deposits were present in 45·9 per cent. of gouty cases, in seven-tenths of which the ear alone was affected. He related two cases to show how greatly the diagnosis may be assisted by the discovery of these minute concretions. Though they are indications of gouty affections, their absence is no proof of its not being gout; they are frequently absent, and patients may suffer gout for many years without their being found. On the other hand, some of the worst cases of deposits have occurred within three or four years from the first attack in the great toe. Another valuable diagnostic symptom is the special great toe affection; and Dr. Garrod shows that, out of a number of cases noticed by him, in 82 per cent. this was present. This symptom is the more valuable, that it seldom, if ever, occurs in rheumatism. The sex, too, is not to be disregarded, as gout is much more common in males than females. Out of a table of cases, only five per cent. were females, which would hardly be the case if the pathology of the two cases were alike. Œdema and subsequent desquamation of the cuticle is almost invariably present in gout, the desquamation showing itself when the inflammation is subsiding. These signs occur very seldom in genuine rheumatism. He also mentioned, among other symptoms, some minor points, which, in conjunction with others, might be of some use in diagnosis—viz., the presence of heart affection in rheumatism from prior attacks of acute disease, the dyspeptic accompaniments of gout, the influence of cold and moisture in inducing rheumatism, and of high living, especially of wine and malt liquors, in bringing on gout, and, lastly, the condition of the blood and blister fluid. In all cases of pure gout, the blood contains an abnormal quantity of uric acid, which is not the case in rheumatism; and in cases where the other symptoms are not characteristically developed, the presence or absence of uric acid in the blood may afford evidence as to the nature of the affection. In the place of abstracting blood, of which only a small quantity is requisite, (an ounce or so,) the examination of the serum produced by a small blister, has the same result.—*Lond. Med. Times and Gazette.*

